# Microstructures and Properties of a Low-Carbon-Chromium Ferritic Stainless Steel Treated by a Quenching and Partitioning Process

**DOI:** 10.3390/ma12101704

**Published:** 2019-05-26

**Authors:** Gang Luo, Huaying Li, Yugui Li, Jinqiang Mo

**Affiliations:** 1 School of Materials Science and Engineering, Taiyuan University of Science and Technology, Taiyuan 030024, China; luoccc2018@163.com; 2 Coordinative Innovation Centre of Taiyuan Heavy Machinery Equipment, Taiyuan University of Science and Technology, Taiyuan 030024, China; 3 School of Material Science and Engineering, Harbin Institute of Technology, Harbin 150001, China; swfx-mjq@163.com

**Keywords:** martensite, retained austenite, ferritic stainless, quenching and partitioning

## Abstract

Low chromium ferritic stainless steel has great potential in automobile structures for improved strength. In this study, quenching and partitioning (Q-P) treatment was applied to a low-carbon-chromium ferritic stainless steel and compared with traditional heat treatment (quenching-tempering [Q-T] and annealing) in terms of microstructure, mechanical properties, corrosion resistance, and deformation of plate. The results show that the quenching and partitioning (Q-P) treatment has a series of advantages over conventional heat treatments (quenching-tempering and annealing). In terms of mechanical properties, it achieves a good match between strength and plasticity by combining the advantages of “soft state” with high elongation resulting from conventional annealing and high strength "hard state” through the traditional quenching-tempering process. The material possesses better crash safety; for the quenching-partitioning (Q-P) process, quenching-tempering process, and annealing process, the production of strength plasticity is about 16 GPa%, 15 GPa%, and 14 GPa%, respectively. The material has low yield strength, high work hardening index (compared with Q-T), a smooth tensile curve, and no yield plateau (compared with annealing), so it has better forming performance and processing surface, and the corrosion resistance has also improved. The pitting potential of the samples produced by the quenching treatment of Q-P and Q-T increased by about 0.2 V, which is about 20% higher than the one by the traditional annealing process.

## 1. Introduction

In recent years, advanced high strength steels (AHSSs) have been in development for automotive applications [1,2,3,4]. AHSSs require a good combination of strength and ductility to meet the weight reduction, reduced fuel consumption and CO_2_ emission, and passenger safety requirements [2,5]. However, AHSSs undergo galvanization treatment for corrosion resistance [6,7,8,9,10], which brings about problems in zinc depletion and environmental pollution [11,12,13,14]. Not only is stainless steel corrosion-resistant, which allows for thinner structures and long-term servicing with low or nearly free maintenance, it is also recyclable and eco-friendly [15,16]. Hence, some efforts have been contributed to the replacement of galvanized AHSS by stainless steels for vehicle body applications (austenitic, ferritic-austenitic, and ferritic stainless steel) [17,18,19,20,21]. Although austenitic and ferritic-austenitic stainless steels have superior corrosion resistance, their applications are limited by relatively high material costs. Comparatively, low chromium ferritic stainless steels contain almost no Ni and low alloy content, and are more economically competitive. However, they are inadequate in their mechanical strength (yield strength is not more than 350 MPa) for automotive structures.

Some research [22,23,24] has shown that low chromium ferritic stainless steel can enhance its strength through martensitic transformation, forming so-called ferrite-martensitic stainless steel (FMSS); however, its plasticity decreases dramatically. In general, during martensitic transformation, a fraction of retained austenite is considered to be an effective means to improve the ductility of high-strength steel where retained austenite increases the toughness and formability by the so-called transformation-induced plasticity (TRIP) effect. However, sufficient retained austenite cannot be produced by conventional quenching and tempering heat treatments. In 2003, Speer [25] proposed a new process, namely “quenching and partitioning” (Q-P), to obtain sufficient retained austenite during martensitic transformation. Different from the traditional quenching and tempering (Q-T) process, the Q-P process utilizes interrupted quenching and austenite stabilization during the partitioning stage with the carbon diffusion from supersaturated martensite to untransformed austenite, and kept to room temperature. The Q-P process has been mainly conducted on C-Si-Mn or C-Si-Mn-Al steels [26,27,28]. Currently, this has begun to be used for medium-chrome ferric stainless steel (Fe-16Cr-0.04C) [29] and martensitic stainless steel (Fe-12Cr-0.1C, Fe-12Cr-0.2C) [30,31,32] for their hardenability. It should be noted that chromium and carbon contribute to the acquisition of retained austenite during Q-P treatment. However, the high content of chromium increases the cost but also reduces the fraction of martensite as well as the strength. The high content of carbon is harmful to the corrosion resistance and weldability.

Therefore, a low-carbon-chromium ferritic stainless steel is chosen in this paper to study the effect of quenching-partitioning treatment on the material from the point of view of microstructure, mechanical properties, corrosion resistance, and plate deformation, and compares it with traditional quenching-tempering and annealing treatments. This study provides experimental support for industrialization.

## 2. Materials and Methods

The experimental material is a low-carbon-chromium stainless steel, and the chemical composition is shown in Table 1. The experimental material was prepared by a ~100 kg vacuum induction melted (VIM) ingot hot-rolled from 100 mm to 6 mm in thickness with a starting temperature of 1150 °C and finishing temperature of 900 °C, which was then annealed at 680 °C for 4 h and cold-rolled to 1.5 mm in thickness.

**Figure 1:**
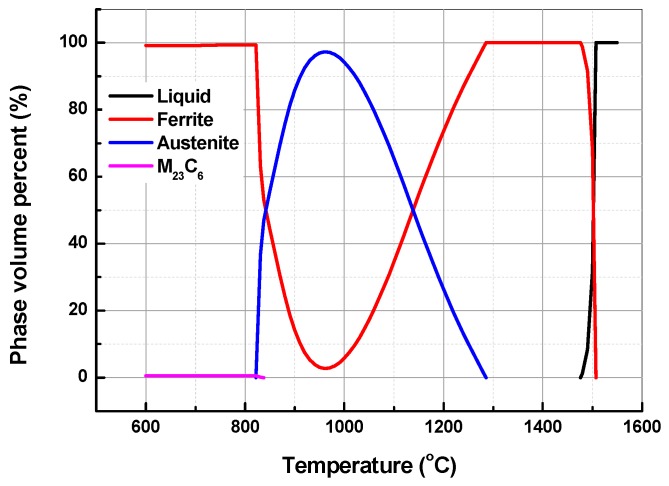
Thermodynamic equilibrium phase diagram of the material under study.

Before the Q-P heat treatment, the phase transformation characteristics of the experimental material were analyzed by means of the equilibrium phase diagram (Figure 1), which was calculated by Thermo-Calc with the TCFE6 data base. The experimental material is in the ferrite single phase region in the high temperature range of 1470 to 1280 °C. From 1280 to 825 °C, it is in the dual phase zone of high temperature for ferrite and austenite, and the content of austenite phase reaches the maximum near 960 °C, which is about 86 wt %. With the decrease of temperature, the proportion of austenite decreases, and the austenite begins to transform into low temperature ferrite until below 825 °C where the austenite is transformed into ferrite and a small amount of carbide Cr_23_C_6_.

**Figure 2:**
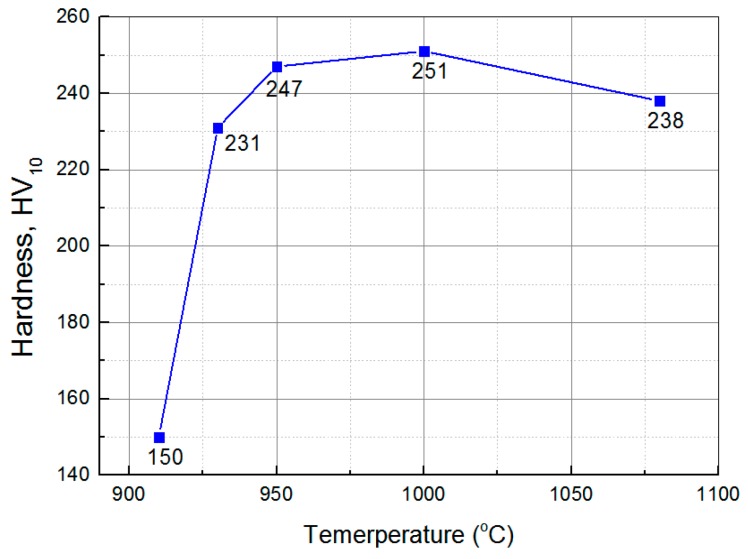
Effect of quenching temperature on hardness of material under study.

In order to obtain a large amount of martensite and retained austenite, the optimum austenitizing temperature should been determined first. After high temperature quenching, low chromium stainless steel is known to undergo phase transformation from austenite to martensite, and the hardness of the material will increase. Therefore, the variation of austenite content with temperature can also be judged by testing the hardness of quenched materials at different temperatures. Figure 2 shows the hardness after quenching at 930–1080 °C. With the increase of temperature, the hardness first increases then decreases. The hardness reaches its maximum value near 1000 °C, with the Vick’s hardness with 10kg load HV_10_ = 251, indicating that the austenite content reaches its maximum at this time. This is slightly different from the equilibrium phase diagram in Figure 1 (austenitizing content is the highest at 960 °C) because element diffusion redistribution is required during austenitizing, and a certain degree of overheating is beneficial to this process. Hence, the austenitizing temperature in this study is 1000 °C.

**Figure 3:**
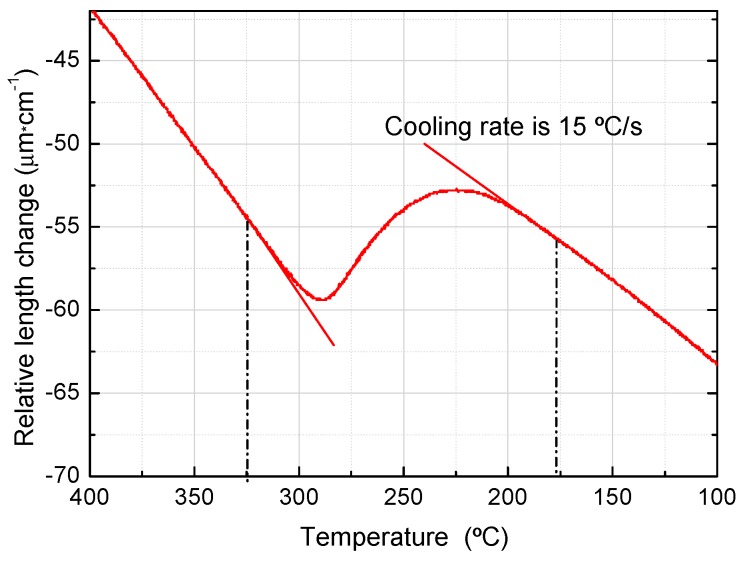
Relative length changes of dilatometric specimen during martensitic transformation.

Figure 3 shows the relationship between the temperature and the expansion caused by martensitic transformation during the cooling process of the hot-rolled material heated at 1000 °C for 5 min and cooled at 15 °C/s. Ms (Martensite Start Temperature) and Mf (Martensite Finish Temperature) can be obtained by tangent method at 325 °C and 172 °C, respectively. Because the quenching temperature Tq is between Ms and Mf in Q-P treatment, the average value of Ms and Mf, i.e., 250 °C, was chosen for this study.

The Q-P treatments were applied to cold-rolled sheets with dimensions of 1.5 mm (thickness) × 80 mm (longitudinal) × 250 mm (traverse). The treatment consisted of partial austenization at 1000 °C for 5 min, quenching (Q) into a salt bath at 250 °C for 10 s, partitioning (P) in another salt bath at 500 °C for 1 min, and final quenching into water at room temperature. The austenization and annealing process was finished in a chamber electric furnace, the quenching process was executed in an internally heated salt bath furnace, and the partition or tempering processes were performed in another salt bath furnace. The salt is a mixture of 50% KNO_3_ and 50% NaNO_3_ melts. In order to ensure high heat transfer rate, 10 kg salt was used.

For comparison, traditional quenching-tempering (Q-T) and annealing treatments were also conducted, respectively. The Q-T treatment consisted of partial austenization at 1000 °C for 5 min, quenching into water at room temperature, tempering in a salt bath at 500 °C for 1 min, and final water quenching. The anneal treatment consisted of heating at 800 °C for 10 min and water-cooling to room temperature. 

The microstructure was observed using a Nova NanoSEM430 scanning electron microscope (FEI, Hillsboro, OR, USA) and a JEM-2100 transmission electron microscope (TEM, JEOL, Tokyo, Japan). The chemical composition of precipitates was identified by energy dispersive X-ray spectrometers (EDXS) under the two microscopes. Thin foil samples were prepared for TEM observation by mechanical thinning and electropolishing within a twin-jet polisher at room temperature. The electrolyte is a solution of 5% HClO_4_ in 95% CH_3_COOH and the jet-polishing voltage is 30 V.

**Figure 4:**
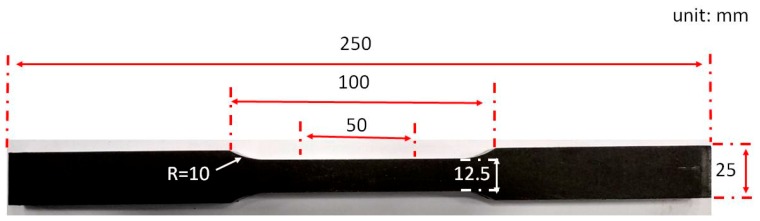
Schema of the flat tensile specimen.

The volume fraction of retained austenite was detected by a X’Pert Pro MPD X-ray diffractometer (XRD, Malvern Panalytical, Malvern, UK) and calculated based on a direct comparison method by integrated intensities of (200)γ, (220)γ, (311)γ, (200)α, and (211)α peaks. Mechanical properties of investigated samples were measured by a Zwick/Roell Z100 tensile tester (Zwick Roell Group, Ulm, Germany). The tensile specimens were cut along the vertical-to-rolling direction and machined into geometries of 12.5 mm parallel width and 50 mm gauge length as shown in Figure 4.

**Figure 5:**
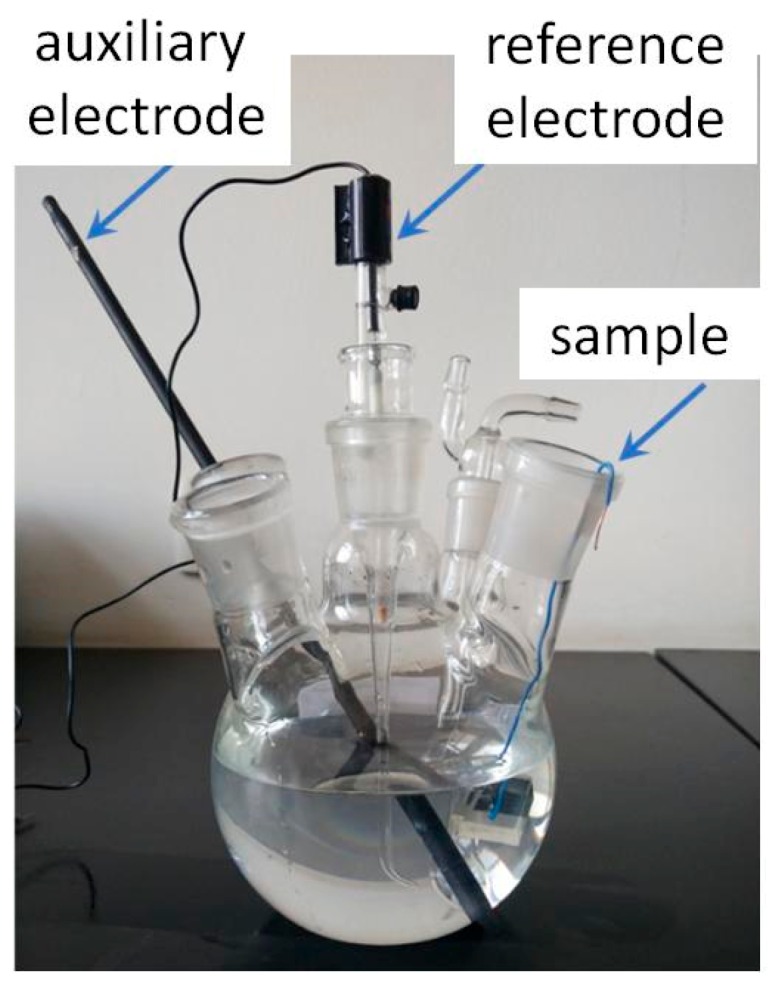
Schema of the experimental set up for the resistance of pitting corrosion.

Pitting corrosion is the main corrosion mode of stainless steel in atmospheric environment. The pitting potential of the sample was tested to characterize the corrosion resistance of the material. The test was completed on a CS350H electrochemical workstation (Wuhan CorrTest Instruments, Wuhan, China). The schema of the experimental set up for the resistance of pitting corrosion is shown in Figure 5. A solution of 3.5 wt % NaCl was used as a substitute of ocean water [33]. Nitrogen was pre-filled in the vessel for half an hour before the experiment to discharge oxygen from the solution. Anodic polarization was carried out with an initial scanning potential of −100mV (vs. Open Circuit Potential) and a scanning rate of 20 mV/min in a 35 °C water bath.

## 3. Results and Discussion

### 3.1. Mircostructure

**Figure 6:**
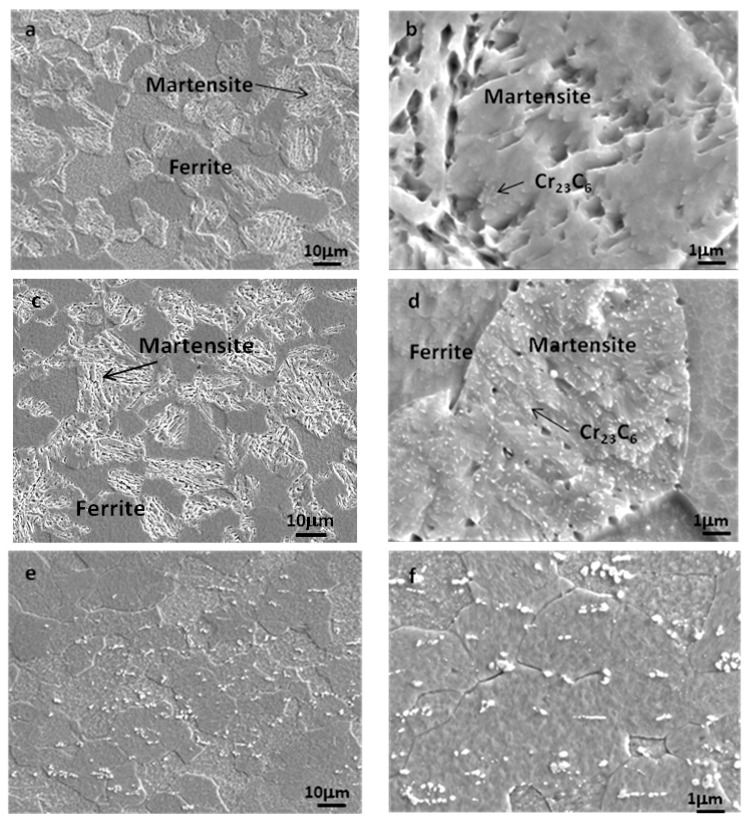
Scanning electron microscopy (SEM) micrographs of the ferrite/martensite complex structure (**a**,**b**) by quenching and partitioning (Q-P) treatment, (**c**,**d**) by quenching and tempering (Q-T) treatment, and (**e**,**f**) by annealing treatment.

Figure 6 shows the SEM photos of samples treated by Q-P (Figure 6a,b), Q-T (Figure 6c,d), and conventional annealing treatments (Figure 6e,f). After Q-P and Q-T treatment, the matrix of the tested material is a mixture of lath martensite and ferrite (see Figure 6a,c). The volume fraction of martensite to ferrite is nearly half to half, which is consistent with the rough estimation from Figure 1 and the Koistinen–Marburger relationship, *f*_m_ = 1 − exp(−0.011(T_s_ − T_q_)), where *f*_m_ is the fraction of austenite transformed into martensite at the quenching temperature T_q_ [34]. The ferrite phase boundary is characterized by an inward depression, which seems to be swallowed by martensite. This is due to the austenite transformation of some ferrites at 1000 °C and the austenite (transformed into martensite after quenching) gradually growing towards ferrite.

**Figure 7:**
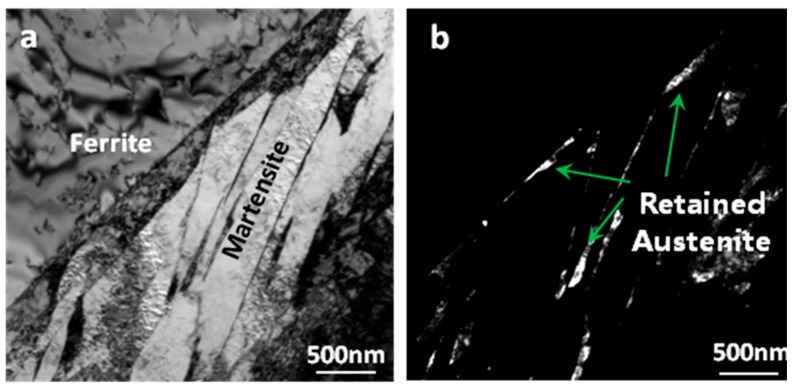
Transmission electron microscopy (TEM) micrographs of Q-P treated specimens: (**a**) ferrite and martensite shown in brightfield and (**b**) retained austenite shown in black field.

Further observation shows that there are a lot of fine carbides precipitated in martensite after Q-T treatment (Figure 6d) but very few carbides in martensite after Q-P treatment (Figure 6b). The results show that Q-P treatment can make the supersaturated carbon element in martensite diffuse into the surrounding untransformed austenite and increase its stability. Therefore, the untransformed austenite changes into retained austenite at room temperature. X-ray diffraction analysis shows that there are weak austenite diffraction peaks in the samples after Q-P treatment. The calculated retained austenite content is about 8%, while the retained austenite can hardly be detected in the samples after Q-T and annealing treatment. Figure 7 shows the transmission electron microscopy (TEM) photos of the samples treated by Q-P. Figure 7a shows that martensite has a lath substructure and high-density dislocation. The dislocation density of ferrite grains around it is low. Retained austenite mainly exists in the form of a thin film between lath martensite or between martensite and ferrite (Figure 7b).

Unlike Q-P and Q-T, the matrix of conventional annealed specimens is ferrite, and there is a lot of Cr_23_C_6_ in the ferrite grains and its boundaries. Because the time of conventional annealing is longer in the precipitation temperature range of 540 °C to *A*_c1_ (Initial Austenitizing Temperature) [35], the total amount of precipitates is obviously more than the former.

### 3.2. Mechanical Properties

**Figure 8:**
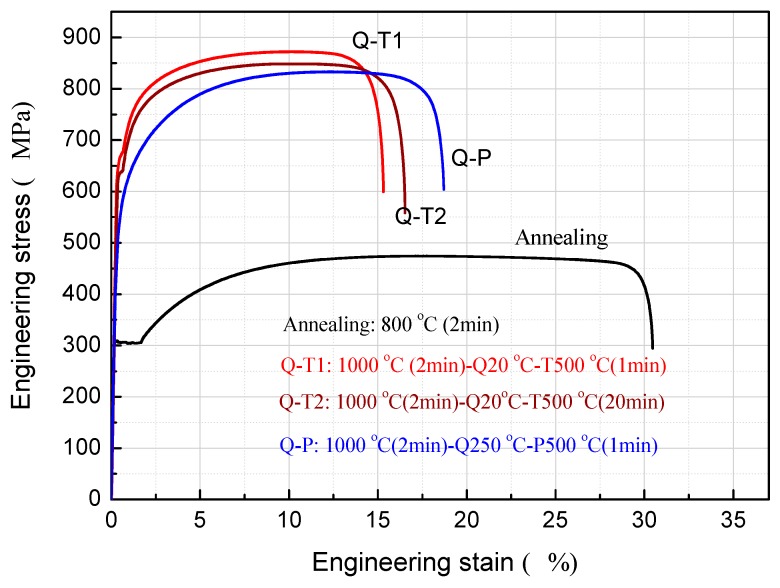
Engineering stress–strain curves of specimens undergoing different treatments.

Figure 8 shows the engineering stress–strain curves of tensile specimens after Q-P, Q-T, and conventional annealing treatments. To make a comprehensive comparison, we also studied Q-T for 20 min, which is long enough to obtain the potential toughness. According to the experimental results, the properties for Q-T with 20 min is still lower than for Q-P. Strength represents the ability of a material to resist external deformation from the point of view of force, and elongation represents the ability of a material to deform without fracture under external force. As an index of energy absorption of materials, the product of strength (R_m_) and total tensile elongation (A), R_m_ × A, has been applied to tailor the steels for automobile application [36,37,38,39]. Table 2 gives the corresponding mechanical properties. The conventional annealing process is characterized by the “soft state” with low strength and high elongation, while the conventional quenching-tempering process is characterized by the “hard state” with high strength and low elongation. The Q-P process has both advantages due to martensite strengthening and the TRIP effect of retained austenite, and achieves a good match between strength and elongation. Compared with the reference [29] (Q-P treated medium-chrome ferritic stainless steel (Fe-16Cr-0.04C)), the tensile strength increased by nearly 80 MPa with similar elongation, which is more conducive to industrial application. The strength increase of the material in this study is due to the more content of mastentic resulting from less Cr.

Further observation of Figure 8 shows that the tensile curves of the samples treated by the traditional heat process (especially annealing) have a “yield plateau”, while the Q-P tensile curves are smooth and the “yield plateau” disappears completely. This is because the matrix produced by traditional processing contains a large number of non-uniformly distributed interstitial atoms, and interstitial atoms aggregate into atom gas clusters at various defects. These atom masses can pin dislocations in the early stage of tensile deformation, hinder the movement of dislocations, and form a “yielding” phenomenon. With the increase of deformation, the pinning effect of interstitial atoms weakens, dislocations move, and materials enter a comprehensive plastic deformation stage. Comparatively, the austenitizing and partitioning process of the Q-P process can enrich interstitial atoms in martensite and retained austenite as far as possible and reduce the interstitial atoms in the matrix significantly. The plastic deformation proceeds continuously in the tensile process and there is no yield plateau. The material, if treated by Q-P, can avoid formation of Ludes band defects in the forming process.

Further compared with the traditional Q-T process, the yield strength of materials from Q-P is lower and the work hardening index is higher, which indicates that materials processed by Q-P has better formability. In addition, from the point of view of vehicle crash safety, materials processed by Q-P have a better strength–plastic product of about 16 GPa%, while the traditional annealing treatment and Q-T treatment correspond to lower strength–plastic product at about 14 GPa% and 15 GPa%, respectively. Q-P treatment can make the material safer during use. The tensile strength and yield strength are two different and essential key functions of the material that can improve crashworthiness. The tensile strength is required by the rigid zone, which is designed to maintain its integrity with minimal intrusion to protect the occupants and the fuel tank, which can easily be achieved by cold working deformation. The yield strength is important especially for complicated fabricated parts like cross members, longitudinal beams, B-pillar reinforcements, sills, and bumper reinforcements, etc, which can be cold-formed by Q-P high strength steel rather than conventional HSS with similar strength levels [36]. The material is required to have high tensile strength in combination with ductility, i.e., high strain hardening regardless of their yield strength [27].

### 3.3. Flatness of Plate

**Figure 9:**
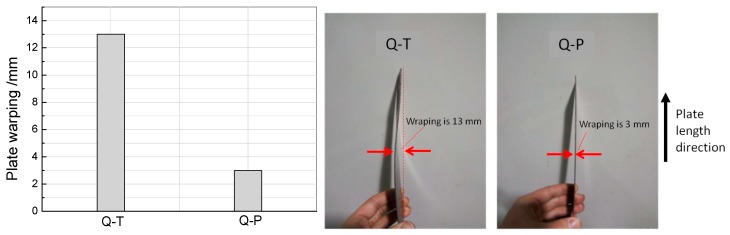
The plate has a better flatness by Q-P treatment than by Q-T treatment. The plate size is 1.5 mm (thickness) × 80 mm (width) × 250 mm (length).

Figure 9 shows the shape of the test steel plate after Q-P treatment and traditional Q-T treatment (size of plate is 1.5 mm (thickness) × 80 mm (width) × 250 mm (length)). The shape corresponding to the traditional Q-T treatment is poor (the warping along the length is up to 13 mm), while that corresponding to the Q-P treatment is better (the warping along length is only 3 mm). Although both the quenching-partitioning process and traditional quenching-tempering process involve martensitic transformation, the internal stress of the steel plate can be further released and is relatively small due to the higher quenching temperature of the quenching-partitioning process. Good shape is conducive to industrial production and application.

### 3.4. Corrosion Resisting Property

**Figure 10:**
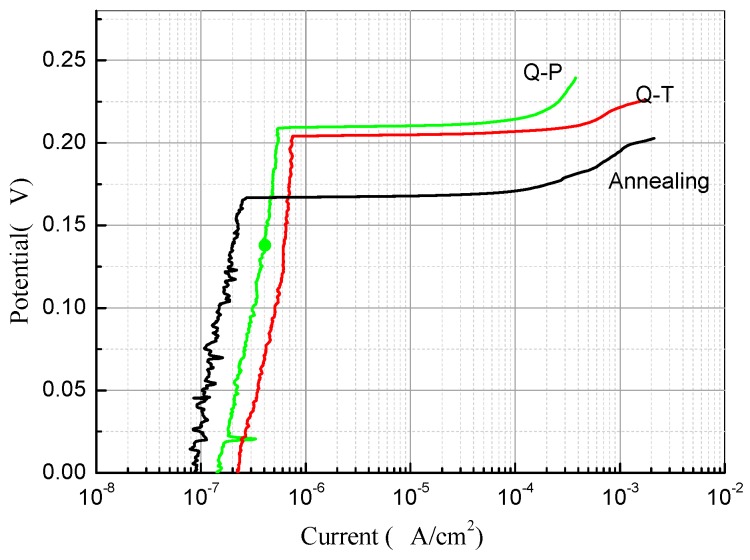
Polarizing curves of the investigated materials by different heat treatments.

Figure 10 shows the polarization curves of Q-P, Q-T, and annealed samples. Because the traditional annealing process will produce a large number of chromium compounds and reduce the chromium content of the matrix, the corresponding corrosion resistance is relatively poor (pitting potential is about 0.17 V). During the austenitizing and quenching treatment of Q-P and Q-T, a large number of Cr_23_C_6_ dissolved and increased the chromium content of the matrix. Therefore, the pitting potential increased by about 0.2 V, which is about 20% higher than the traditional annealing process. At the same time, the pitting corrosion resistance of Q-P treated specimens is better because less chromium compounds are precipitated during tempering than that for Q-T treated specimens (see Figure 6).

### 3.5. Comparing with the AHSS

The results show that the mechanical properties of low chromium ferritic stainless steel by quenching and partitioning treatment are close to 780 MPa grade AHSS, which is the most widely used steel at present. Compared with TRIP and Q-P steels which have been developed for 60 years [40] and 15 years [25], respectively, the mechanical properties of Q-P low chromium ferritic stainless steel have reached 70% of the maximum values of TRIP and Q-P steels. However, the eorrosion resistance is greatly improved compared to TRIP and Q-P steels. The low-carbon-chromium ferritic stainless steel by Q-P has found widespread use in wet sliding abrasion conditions and in aqueous environments involving exposure or immersion, such as the cargo body for coal transportation.

## 4. Conclusions

After quenching-partitioning (Q-P) treatment, the test material has a series of advantages:

(1) Combination of conventional annealing “soft state” with high elongation and traditional quenching-tempering process “hard state” with high strength. It achieves a good match between strength and plasticity, and has better impact safety (for the quenching-partitioning [Q-P] process, quenching-tempering process, and annealing process, the production of strength plasticity is about 16 GPa%, 15 GPa%, and 14 GPa%, respectively).
(2) The material has low yield strength, high work hardening index (compared with Q-T), smooth tensile curve, and no yield plateau (compared with annealing), so it has better forming performance, and finally, the corrosion resistance of the material is improved by 20% in terms of pitting potential (compared with annealing).

## Figures and Tables

**Table 1:** Chemical compositions of experimental material.

C	Si	Mn	P	S	Cr	Ni	N
0.030	0.65	0.24	0.023	0.001	12.97	0.08	0.018

**Table 2:** Properties of specimens processed through different treatments, with the corresponding uncertainty in parentheses: yield strength (±10 MPa), tensile strength (±5 MPa), elongation (±0.5%), and work hardness index (±0.05).

Heat Treatment	Yield Stress Rp_0.2_/MPa	Tensile Strength R_m_/MPa	Elongation A_50_/%	Work Hardening Index	Strength-Elongation Production R_m_ × A_50_/GPa %
Q-P	544	833	18.7	0.16	15.6
Q-T1	670	872	15.3	0.11	13.3
Q-T2	635	848	16.5	0.11	14.0
Annealing	304	474	30.5	0.29	14.5

Q-P (quenching and partitioning): 1000 °C, 5 min; 250 °C; 500 °C, 1 min. Q-T1 (quenching and tempering): 1000 °C, 5 min; 20 °C; 500 °C, 1 min. Q-T2 (quenching and tempering): 1000 °C, 5 min; 20 °C; 500 °C, 20 min. Annealing: 800 °C, 10 min.
